# Safety and Efficacy of Convalescent Plasma to Treat Severe COVID-19: Protocol for the Saudi Collaborative Multicenter Phase II Study

**DOI:** 10.2196/23543

**Published:** 2020-10-02

**Authors:** Mohammed Albalawi, Syed Ziauddin Ahmed Zaidi, Nawal AlShehry, Ahmed AlAskar, Abdul Rehman Zia Zaidi, Rania Nagib Mohammed Abdallah, Abdul Salam, Ahmed AlSagheir, Nour AlMozain, Ghada Elgohary, Khalid Batarfi, Alia Alfaraedi, Osamah Khojah, Rehab Al-Ansari, Mona Alfaraj, Afra Dayel, Ahmed Al Bahrani, Arwa Nabhan Abdelhameed, Hind Alhumaidan, Jawaher M Al-Otaibi, Ghazala Radwi, Abdulrahman Raizah, Hind Shatry, Sara Alsaleh, Hazzaa AlZahrani, Hani Al-Hashmi

**Affiliations:** 1 Department of Internal Medicine College of Medicine Taibah University Madinah Saudi Arabia; 2 Department of Adult Hematology/Blood & Marrow Transplant King Fahad Medical City Riyadh Saudi Arabia; 3 King Abdullah International Medical Research Center King Saud bin Abdulaziz University for Health Sciences King Abdulaziz Medical City Riyadh Saudi Arabia; 4 Department of Medicine King Fahad Medical City Riyadh Saudi Arabia; 5 Department of Pulmonary and Critical Care Medicine King Fahad Medical City Riyadh Saudi Arabia; 6 Department of Epidemiology and Biostatistics King Fahad Specialist Hospital Dammam Saudi Arabia; 7 Department of Hematology and Oncology Johns Hopkins Aramco Healthcare Dhahran Saudi Arabia; 8 Department of Blood Bank King Saud University Medical City King Saud University Riyadh Saudi Arabia; 9 Oncology Center King Saud University Medical City King Saud University Riyadh Saudi Arabia; 10 Transfusion Medicine Services Department of Pathology & Laboratory Medicine King Abdulaziz Medical City Riyadh Saudi Arabia; 11 King Saud University Riyadh Saudi Arabia; 12 Department of Medicine King Fahad Military Medical Complex Dhahran Saudi Arabia; 13 Hematology Laboratory Section of Medical Laboratory Department Qatif Central Hospital Qatif Saudi Arabia; 14 Department of Hematopathology King Fahad Specialist Hospital Dammam Dammam Saudi Arabia; 15 Department of Transfusion Medicine and Stem Cell Processing King Fahad Specialist Hospital Dammam Saudi Arabia; 16 Department of Internal Medicine Imam Abdulrahman Bin Faisal University Al-Khobar Saudi Arabia; 17 Department of Pathology and Laboratory Medicine King Faisal Specialist Hospital and Research Center Riyadh Saudi Arabia; 18 Department of Infectious Diseases King Faisal Specialist Hospital and Research Center Riyadh Saudi Arabia; 19 Department of Hematology University of Alberta Edmonton, ON Canada; 20 Department of Hematology and Bone Marrow Transplantation Section King Faisal Specialist Hospital and Research Center Riyadh Saudi Arabia; 21 Adult Hematology & Stem Cell Transplantation Department Oncology Centre King Fahad Specialist Hospital Dammam Saudi Arabia

**Keywords:** coronaviruses, SARS-CoV-2, COVID-19, antibodies, convalescent plasma, treatment, immunology, feasibility, safety, efficacy, infectious disease

## Abstract

**Background:**

The COVID-19 pandemic is expected to cause significant morbidity and mortality. The development of an effective vaccine will take several months to become available, and its affordability is unpredictable. Transfusion of convalescent plasma (CP) may provide passive immunity. Based on initial data from China, a group of hematologists, infectious disease specialists, and intensivists drafted this protocol in March 2020.

**Objective:**

The aim of this study is to test the feasibility, safety, and efficacy of CP in treating patients with COVID-19 across Saudi Arabia.

**Methods:**

Eligible patients with COVID-19 will be recruited for CP infusion according to the inclusion criteria. As COVID-19 has proven to be a moving target as far as its management is concerned, we will use current definitions according to the Ministry of Health (MOH) guidelines for diagnosis, treatment, and recovery. All CP recipients will receive supportive management including all available recommended therapies according to the available MOH guidelines. Eligible CP donors will be patients with COVID-19 who have fully recovered from their disease according to MOH recovery criteria as detailed in the inclusion criteria. CP donors have to qualify as blood donors according to MOH regulations except for the history of COVID-19 in the recent past. We will also test the CP donors for the presence of SARS-CoV-2 antibodies by a rapid test, and aliquots will be archived for future antibody titration. Due to the perceived benefit of CP, randomization was not considered. However, we will compare the outcome of the cohort treated with CP with those who did not receive CP due to a lack of consent or lack of availability. In this national collaborative study, there is a likelihood of not finding exactly matched control group patients. Hence, we plan to perform a propensity score matching of the CP recipients with the comparator group patients for the major characteristics. We plan to collect demographic, clinical, and laboratory characteristics of both groups and compare the outcomes. A total sample size of 575 patients, 115 CP recipients and 460 matched controls (1:4 ratio), will be sufficient to detect a clinically important hospital stay and 30-day mortality difference between the two groups with 80% power and a 5% level of significance.

**Results:**

At present, patient recruitment is still ongoing, and the interim analysis of the first 40 patients will be shared soon.

**Conclusions:**

In this paper, we present a protocol for a national collaborative multicenter phase II study in Saudi Arabia for assessing the feasibility, safety, and potential efficacy of CP in treating patients with severe COVID-19. We plan to publish an interim report of the first 40 CP recipients and their matched comparators soon.

**Trial Registration:**

ClinicalTrials.gov NCT04347681; https://clinicaltrials.gov/ct2/show/NCT04347681

**International Registered Report Identifier (IRRID):**

PRR1-10.2196/23543

## Introduction

### Background

The COVID-19 pandemic, caused by SARS-CoV-2, is a major health and economic concern worldwide due to its morbidity and mortality. Coronaviruses are a large family of RNA viruses that cause illnesses ranging from the common cold to more severe diseases such as Middle East respiratory syndrome–related coronavirus (MERS-CoV) and severe acute respiratory syndrome–related coronavirus (SARS-CoV) [1]. The new strain of coronavirus identified in December 2019 in Wuhan City, Hubei Province of China was called the 2019 novel coronavirus. The International Committee on Taxonomy of Viruses determined that SARS-CoV-2 is the same species as SARS-CoV. The World Health Organization (WHO) has named the disease associated with SARS-CoV-2 infections COVID-19.

Clinical features of SARS-CoV-2 infection typically include fever and respiratory symptoms like cough and shortness of breath; in severe cases, the infection can cause pneumonia, severe acute respiratory distress syndrome (ARDS), kidney failure, and even death. SARS-CoV-2 has a higher transmission rate with an approximate fatality rate of 3% [2]. The final diagnosis of SARS-CoV-2 infection depends on laboratory detection of the SARS-CoV-2 viral RNA by real-time reverse transcription–polymerase chain reaction (rRT-PCR) [1-4].

The concept of using convalescent plasma (CP) is not new since it has been tried in limited numbers of patients during recent viral crises, including the 2003 severe acute respiratory syndrome epidemic, the 2009 “swine flu” epidemic, and the 2012 outbreak of Middle East respiratory syndrome [5]. CP treatment reduced mortality in patients with severe pandemic influenza A 2009 virus infection [6]. Patients with a resolved viral infection typically develop a polyclonal antibody immune response to different viral antigens. Some of these polyclonal antibodies will likely neutralize the virus and prevent new rounds of infection. Patients who recovered from COVID-19 can donate plasma, and then, this plasma can be transfused into patients who are actively infected [7]. Indeed, the same rationale was used in the treatment of several patients with Ebola with convalescent serum during the outbreak in 2014-2015 [8].

Therefore, CP therapy is expected to improve the clinical, laboratory, and radiological features of the patients severely affected by COVID-19. Decreasing the morbidity and mortality of COVID-19 in a cost-effective manner, leading to improved quality of health care and self-sufficiency in the treatment of serious diseases affecting the masses, is in line with the top priorities in Vision 2030 of the Kingdom of Saudi Arabia.

### SARS-CoV-2–Specific Immunoglobulins Containing CP

Among the most attractive intuitive options, during this fast-kinetic pandemic, is treating the patients who are sick with COVID-19 with SARS-CoV-2–specific immunoglobulins found in patients who have fully recovered from COVID-19 and are considered no longer infected. We know from prior research that antibodies against viral antigens render people immune, but we do not know yet how long the immunity will last. However, Zhao et al [9] showed seroconversion in 173 patients with COVID-19 appeared for total antibody, immunoglobulin M (IgM), and immunoglobulin G (IgG) at 11, 12, and 14 days [9]. The presence of antibodies was <40% in the first 7 days and then rapidly increased to 100%, 94.3%, and 79.8% for antibodies, IgM, and IgG, respectively, by day 15. In contrast, viral RNA decreased from 66.7% before day 7 to 45.5% in days 15-39. Moreover, a higher titer of antibodies was independently associated with clinically worse COVID-19 (*P*=.006) [9].

### Antibodies Detection by a Rapid Serological Method and Their Kinetics

In those patients who have passed the viremic phase, the presence of antibodies is highly desirable and provides evidence of the immunity to combat COVID-19 (Table 1). Fortunately, the Saudi Food and Drug Authority (SFDA) has recently approved a needed rapid test kit made by BIOZEK company and other brands in the Saudi market that qualitatively detect IgG and IgM antibodies against SARS-CoV-2 from whole blood, serum, or plasma using a single-use cassette. This kit uses lateral flow chromatographic immunoassay and can produce results within 10 minutes. The combination use of the IgM and IgG tests can reflect virus infection and the immune status of the body effectively (Table 1).

**Table 1:** How to interpret the results of PCR and antibody results.^a^

Test results			Clinical Significance	
PCR^b^	IgM^c^	IgG^d^		
✓^e^	—^f^	—	The patient may be in the window period of infection.	
✓	✓	—	The patient may be in the early stage of infection.	
✓	✓	✓	The patient is in the active phase of infection.	
✓	—	✓	The patient may be in the late or recurrent stage of infection.	
—	✓	—	The patient may be in the early stage of infection. PCR result may be false-negative.	
—	—	✓	The patient may have had a past infection and has recovered.	
—	✓	✓	The patient may be in the recovery stage of an infection, or the PCR result may be false-negative.	

^a^Adapted from [10,11].

^b^PCR: polymerase chain reaction.

^c^IgM: immunoglobulin M.

^d^IgG: immunoglobulin G.

^e^Indicates a positive result.

^f^Indicates a negative result.

### Role of CP in Treating Severe COVID-19

Casadevall and Pirofski [12] suggested that convalescent sera from individuals with COVID-19 may be an option for treating the highest-risk patients with COVID-19 and possibly for prophylaxis of infection in individuals at high risk of COVID-19. This passive antibody administration concept to prevent disease is already used in patients exposed to hepatitis B and rabies viruses, and to prevent severe respiratory syncytial virus disease in high-risk infants [12]. The proposed use of convalescent sera in the COVID-19 epidemic would rely on preparations of high titers of neutralizing antibodies against SARS-CoV-2.

In the first pilot Chinese study reported by Duan et al [13], CP therapy in 10 patients showed a potential therapeutic effect and low risk in the treatment of patients with severe COVID-19. One dose of CP with a high concentration of neutralizing antibodies can rapidly reduce the viral load and tends to improve clinical outcomes [13]. The optimal dose and treatment time point, as well as the definite clinical benefits of CP therapy, need to be further investigated in randomized clinical studies. Out of 40 donors (recovered patients with COVID-19), 39 showed a high antibody titer of at least 1:160. After receiving CP therapy, 9 out of 10 recipients were found to have neutralizing antibody titers >1:640 [13]. Although small, it is a pivotal study to prove the safety and efficacy of CP therapy. All patients showed an increase in oxygen saturation within 3 days. Other parameters that improved were increased absolute lymphocyte counts (ALCs) and decreased C-reactive protein (CRP). Varying degrees of resolution of lung lesions were also seen on radiological examinations within 7 days. In 7 patients who previously had viremia, the viral load was undetectable after transfusion [13].

### Currently Available Therapeutic Options for COVID-19

**Figure 1:**
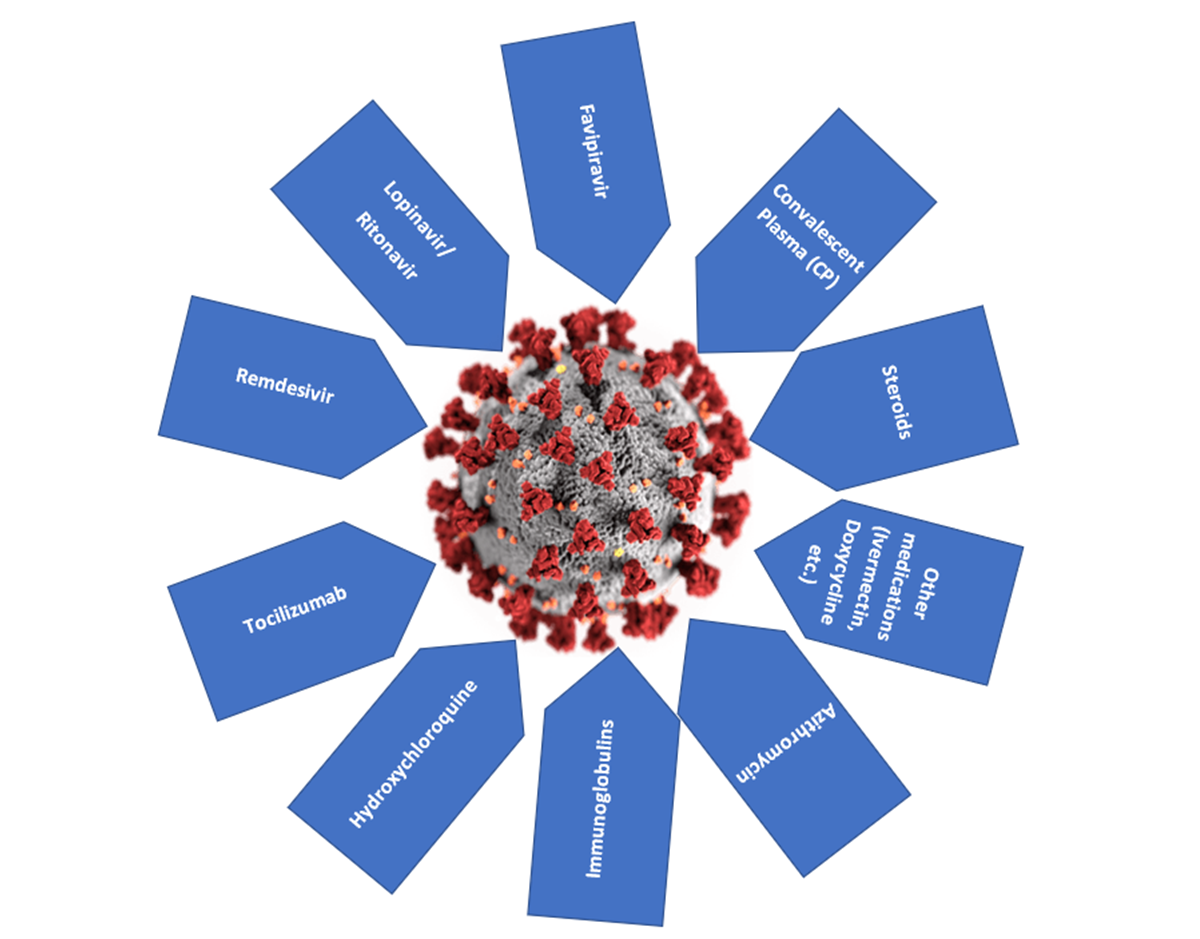
Some therapeutic options for treating COVID-19.

According to the WHO, the management of COVID-19 has mainly focused on infection prevention, case detection, monitoring, and supportive care, although there are reports on the efficacy of new potential therapeutic agents (Figure 1). However, no specific anti–SARS-CoV-2 treatment is recommended because of conflicting evidence. Evidence shows that CP from patients who have recovered from viral infections can be used as a treatment without the occurrence of severe adverse events (SAE). Therefore, it might be worthwhile to test the safety and efficacy of CP transfusion in patients infected with SARS-CoV-2 [14].

Our study aims to test the feasibility, safety, and efficacy of CP in treating patients with COVID-19 across Saudi Arabia.

## Methods

This is a national, phase II, multicenter trial evaluating the safety and potential efficacy of CP to treat severe COVID-19 and patients at high risk of developing severe COVID-19. Detailed bilingual informed consent forms (ICFs) approved by the Ministry of Health (MOH) and institutional review boards (IRBs) will be used for both CP donors and CP recipients.

### Time of the Study

The proposed duration of 3 months was considered ideal for recruiting the first 40 CP recipients for our proposed prospective study. If interim analysis shows the benefit of CP, we will increase the sample size and extend the trial period.

### Inclusion Criteria

The inclusion criteria for recipients is as follows:

- 18 years or older
- Patients with COVID-19 confirmed by positive rRT-PCR test for SARS-CoV-2 “using one of the SFDA approved kits used in the Kingdom of Saudi Arabia” as per current MOH guidelines
- Must have required intensive care unit (ICU), severe, or immediately life-threatening care:
  Patient requiring ICU care or admission
  Severe disease is defined as dyspnea, respiratory frequency≥30/min, blood oxygen saturation≤93%, the partial pressure of arterial oxygen to fraction of inspired oxygen ratio<300, or lung infiltrates>50% within 24-48 hours.
  Life-threatening disease is defined as respiratory failure, septic shock, or multiple organ dysfunction or failure.

The inclusion criteria for the donors was as follows:

- 18 years or older
- Prior confirmed COVID-19 diagnosis as per current MOH guidelines
- Complete clinical recovery from COVID-19 before donation (at least 14 days from the last SARS-CoV-2 negative polymerase chain reaction or 28 days from the initial symptoms) [15-18]
- All MOH criteria for blood donation will be followed.
- All transfusion transmissible infections markers on the donor’s blood are negative as per current MOH routine blood donor screening regulations.
- Positive rapid serology test for antibodies (IgG) against SARS-CoV-2 indicating immunity against COVID 19

### Exclusion Criteria

The exclusion criteria for recipients was a negative or nonconclusive COVID-19 rRT-PCR test for SARS-CoV-2, mild symptoms, and hospitalization not requiring ICU care or admission. The exclusion criteria for donors was being unfit for blood donation or a multiparous or pregnant female.

### Collection of CP Infusion for Treatment of COVID-19

As antibody kinetics show IgG levels are highest around day 28 and decline around day 42 to complete disappearance in many months, we will try to get plasma donation from fully recovered patients soon after day 28 of the onset of symptoms (that usually last for 7-10 days). For the recovery definition, we will continue to follow the MOH’s current precautionary protocol to prevent the spread of the virus causing COVID-19 that requires blood donor abstinence from donation for 28 days from exposure [17,18].

**Figure 2:**
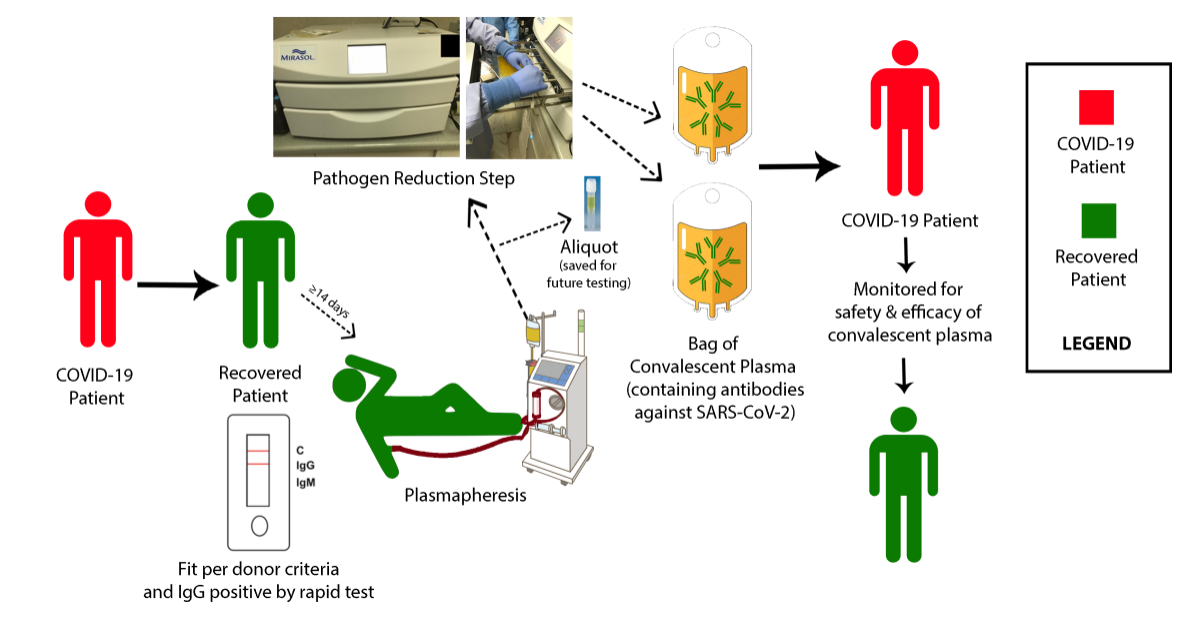
Logistic cycle of convalescent plasma procurement from donor, processing, and infusion to patient with COVID-19. IgG: immunoglobulin G; IgM: immunoglobulin M.

Plasmapheresis to collect plasma from the donors (using Trima, Hemonetics, or alike machines) is a commonly used procedure. Donors are required to be in an acceptable health state and pass through a multistep screening (donor history questionnaire, vital signs check, laboratory tests, etc) before CP donation. The arrangement for plasmapheresis and collection will start after obtaining the donor’s informed consent. The collected plasma will undergo an additional safety step of pathogen reduction using Mirasol or Intercept Pathogen Reduction Technology, which is SFDA and CE (Conformité Européenne) approved (Figure 2). The Mirasol system uses vitamin B2 (riboflavin) and ultraviolet light. Mirasol-treated fresh frozen plasma (FFP) maintains a good quality of therapeutic proteins as demonstrated in multiple external validation studies [19-21]. After passing through the pathogen reduction system, the CP will be sent to the COVID-19 recipients, or it can be stored in a dedicated FFP freezer at ≤–18 °C. The shelf life of frozen CP should be similar to normal FFP according to the storage conditions (1 year at ≤–18 °C or 24 hours at 1-6 °C). The CP units will be labeled, stored, and shipped as per Central Board for Accreditation of Healthcare Institutes (CBAHI), American Association of Blood Banks (AABB), and Joint Commission International guidelines for blood products handling and management.

CP will be used only for eligible patients who have COVID-19. The common side effects of FFP transfusion include side effects of blood product transfusion (eg, allergic or febrile reactions, transfusion-related acute lung injury [TRALI], and transfusion-associated circulatory overload [TACO]) while the infectious risk is minimal. Plasma volume to be collected from each donor can be up to 15% of the total blood volume (TBV; TBV = weight in kg x 70 ml). For example, from a CP donor who weighs 65 kg, we can collect up to ~682 ml plasma (15 / 100 x 65 x 70). CP donors can donate more than once as per the regulations of the CBAHI and AABB, which allows healthy donors to donate plasma twice in a month up to a maximum of 24 donations in a year. Special International Society of Blood Transfusion labels will be affixed on the plasma bags indicating COVID-19 CP (Figure 2).

### ICF

The ICF will be used for both the CP donor and the recipient (Approved Donor ICF [Arabic and English] and Approved Recipient ICF [Arabic and English]).

### Transfusion of CP for Treatment of COVID-19

**Figure 3:**
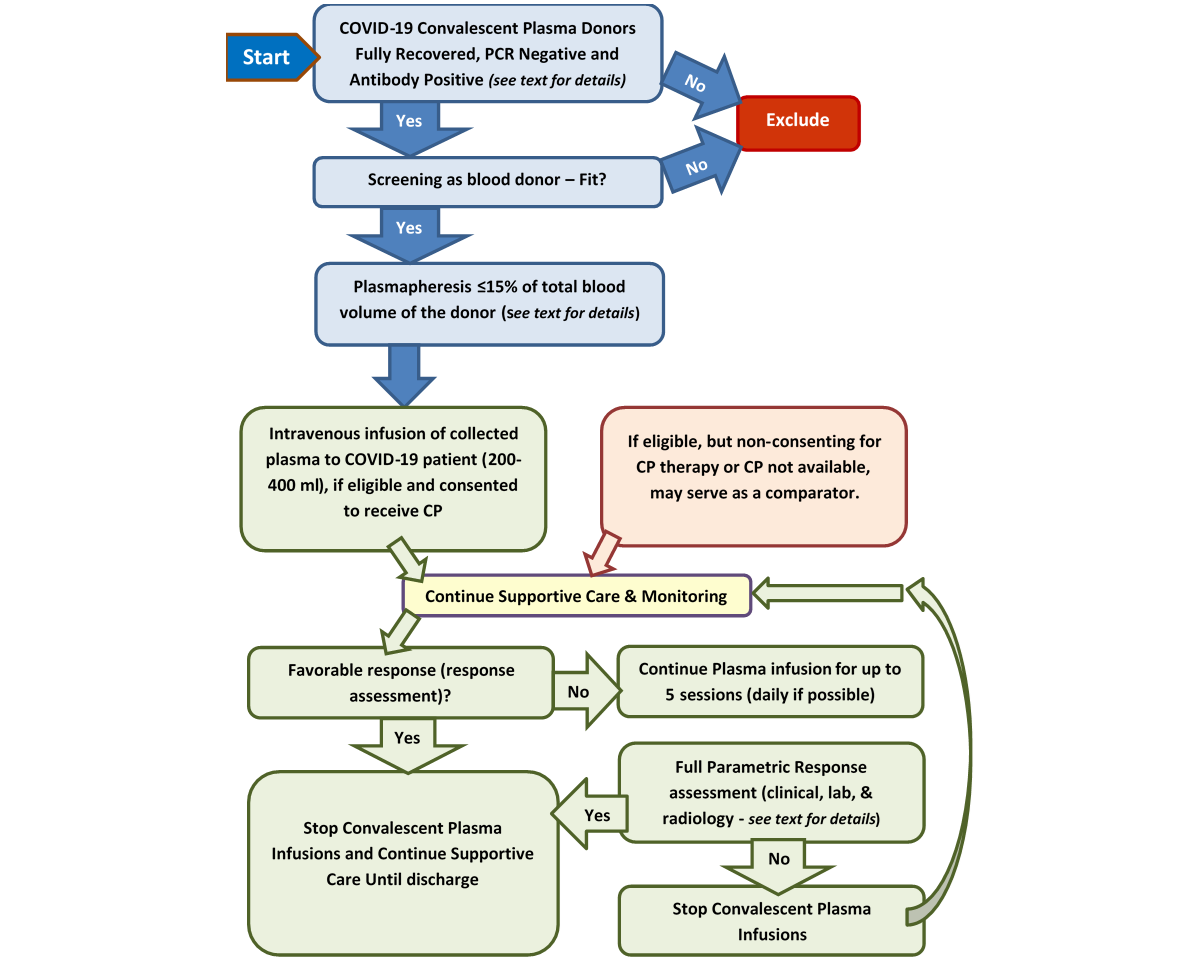
Schematic for the proposed process of CP donation and infusion. CP: convalescent plasma; PCR: polymerase chain reaction.

After obtaining informed consent, eligible patients who have severe COVID-19 and have not recovered yet will be infused with the donated CP 300 ml (200-400 ml/treatment dose) at least once and, if needed, daily for up to 5 sessions (Figure 3).

Other supportive and therapeutic measures should continue according to the locally approved protocols with due diligence. Patients will be monitored after FFP transfusion for the usual side effects of blood product transfusion (eg, allergic or febrile reactions, TRALI, and circulatory overload). We will then assess the response after the infusion of the plasma in these patients as detailed in the Response Assessment section.

As with other plasma therapies, attention should be given to ABO compatibility. For plasma selection, we will consider ABO compatibility (Table 2) regardless of Rh status. To minimize the risk of TRALI, preference will be given to plasma from male donors and nulliparous women.

**Table 2:** ABO group selection order for transfusion of plasma.^a^

Patient’s ABO group		Fresh frozen plasma
**O**		
	First choice	O
	Second choice	A or B
	Third choice	AB
**A**		
	First choice	A
	Second choice	AB
	Third choice	B^b^
**B**		
	First choice	B
	Second choice	AB
	Third choice	A^b^
**AB**		
	First choice	AB
	Second choice	A^b^
	Third choice	B^b^

^a^Adapated from [22].

^b^Tested and negative for high titer anti-A and/or anti-B (should be less than 1:64).

### Data Collection

Clinical information of all enrolled patients will be retrieved from the hospital electronic or paper records system, including the following:

- Baseline demographic data
- Days of illness duration
- Presenting symptoms
- Radiological findings (chest x-ray and computed tomography [CT] scan chest, if possible) on the day of hospital admission; on the day of ICU admission; on the day of CP infusion (day 0); and on days 3, 7, 14, and 30
- Laboratory infectious marker testing results like respiratory, urinary, or blood culture
- Laboratory inflammatory markers: CRP (day of CP infusion [day 0] and then on days 3, 7, 14, and 30)
- Application of assisted mechanical ventilation and their modes, intranasal oxygen inhalation, number of days of intubation, or nasal oxygen support
- Medication regimen (eg, hydroxychloroquine, azithromycin, any antiviral therapies, steroids, tocilizumab)
- Complications including acute renal failure, acute coronary syndrome, myocarditis, ARDS, gastrointestinal complication, and nosocomial infection
- The SARS-CoV-2 RNA from the serum sample will be monitored during treatment and at day 14 of recovery or discharge, whichever is later.

We plan to test for all of the following parameters for patients with COVID-19:

- Complete blood count (CBC) differential to include percent and ALC, and percent and absolute neutrophil count
- Chemistry panel to include total protein, albumin, lactate dehydrogenase, alanine aminotransferase and aspartate aminotransferase, and procalcitonin
- Cardiac biomarkers (eg, cardiac troponins)
- Creatine phosphokinase
- Ferritin
- Full coagulation profile to include partial thromboplastin (PT), activated PT time, fibrinogen, and D-dimer
- CRP
- Oxygen saturation
- Radiological examination
- ABO RhD grouping and antibody screening
- rRT-PCR test for SARS-CoV-2
- Test for IgG and IgM antibodies against SARS-CoV-2

CP donors will essentially undergo routine blood donation processes such as donor history questionnaire, clinical examination, and testing for the infectious marker (serology and nucleic acid testing methods) along with ABO RhD grouping, antibody screening, and CBC. They will also undergo a test for IgG and IgM antibodies against SARS-CoV-2, which should be positive for IgG.

Data collection forms have been developed to collect data for CP donors, recipients, and controls (comparators).

### Response Assessment

The response assessment includes daily clinical assessment by a physician; vital signs including temperature, blood pressure, respiratory rate, and heart rate; oxygen saturation; oxygen requirement; ventilator requirement and the modes employed; inotrope medications requirement; CBCs, liver function tests, urea, creatinine, and electrolytes daily; Apache score; Sequential Organ Failure Assessment score; fluid balance; x-ray or CT changes, repeated every 3-5 days; organs functions assessment; plasma doses and frequency requirement; transfusion-related side effects including TRALI, TACO, etc; and the SARS-CoV-2 RNA will be tested on recovery (or deterioration to determine alternative etiology).

### Study Endpoint and Outcome Measures

Our primary endpoints are ICU (or designated area for critical patients) length of stay; safety of CP; and reporting of SAE such as anaphylaxis, TRALI, and TACO.

Our secondary endpoints will include number of days on mechanical ventilation, 30-day mortality, and days to clinical recovery as defined by the MOH.

### Study Population

A total sample size of 575 patients, 115 CP recipients and 460 matched controls (1:4 ratio), will be sufficient to detect a clinically important difference of 11.6% between two groups (CP recipients vs matched controls) in 30-day mortality using a two-tailed *z* test of proportions and chi-square test with 80% power and a 5% level of significance. This 11.6% difference represents a 12.4% mortality in the CP recipient group and 24.4% mortality in the matched control patients [23].

The treatment group (CP recipients group) will have 115 patients who have COVID-19 but have not recovered yet as per the inclusion criteria. The control group (comparator group) will have 460 patients who are either not consenting to receive CP or those who will not be able to receive CP due to nonavailability to compare the efficacy of the CP. Control group patients will be subjected to propensity score (PS) matching based on age, gender, diabetes mellitus (DM), hypertension (HTN), and intubation.

### Statistical Analysis

Descriptive and inferential statistics will be used to characterize the study sample and test the hypotheses. Descriptive results for all quantitative variables such as age will be presented as mean (SD; for normally distributed data) or median with interquartile range (for data not normally distributed), while numbers (percentage) will be reported for all qualitative variables such as gender.

To assess the independent effect of CP transfusion safety and survival, we will conduct a PS-matching (based on age, gender, DM, HTN, and intubation) analysis. Among the predictors, exact matching will be enforced to achieve the balance for all predictors between the plasma and control groups.

The bivariate analysis will be performed using independent sample *t* test, Mann-Whitney *U* test, Pearson chi-square test, or Fisher exact test whenever appropriate to compare the demographic characteristics (eg, age, gender, nationality) and clinical characteristics (improvement in oxygenation, laboratory parameters, radiological findings, complications, and length of hospital stay) between those who will receive the CP and those who will not receive this therapy.

The multiple binary logistic regression model will be used to assess the effect of CP transfusion on 30-day mortality after adjusting for potential confounding factors compared to matched controls patients. The adjusted odds ratio and 95% confidence interval for the adjusted odds ratio will be reported. The Hosmer-Lemeshow goodness-of-fit statistics will be used to determine whether the model adequately describes the data.

The time-to-event analysis will be measured from the date of diagnosis. The overall survival at 30 days and 3 months will be evaluated using the Kaplan-Meier estimator and compared between the two groups (plasma recipients vs matched control patients) using the log-rank test. A Cox proportional hazard model will also be used to estimate the hazard ratio for in-hospital 30-day mortality for the plasma group compared with matched control group patients after adjusting for potential confounding factors. In addition, interactions between CP administration and all the predictors will be tested to see if the plasma effects will be the same in subgroups. A *P*<.05 (two-tailed) will be considered statistically significant. All statistical analyses will be performed using SPSS version 24 (IBM Corp).

### Interim Analysis

An interim analysis will be performed after enrolling 40 CP recipients and 40 PS-matched controls. A similar statistical analysis will be performed as previously described.

### Monitoring and Safety

Plasma infusion is a routine practice in health care facilities. All known adverse events and SAE of CP infusion, such as anaphylaxis, TRALI, and TACO, will be collected as per the SFDA reporting standard. SAE or death of a study participant due to any cause will be reported by the study team to the IRB chairman and principal investigator within 24 hours of the event.

### Confidentiality Statement

All subject-related personal information will be saved in password-protected files, which will only be accessed by the study research team. All data will be archived in our archiving facility within the hospital once the study has come to an end. This will be in accordance with the standard requirement for the clinical trial archival.

### Patient and Family Education, and Donor Recruitment Strategy

A variety of methods will be used to recruit CP donors for the study. These include referrals of the recovered patients from various hospitals, dissemination of messages to the public through social media platforms, and a website set up specifically for the study for general information and communication [24].

We will be using the following Twitter account: @Plasma4CovidKSA for public information only. Approved videos will be used on the website and Twitter account. In addition, multilingual advertisement statements and links to the videos by international physicians may be shared with non-Arabic speaking patients. Detailed bilingual ICFs approved by the MOH and IRBs will be used for both CP donors and CP recipients.

## Results

We are still collecting data and recruiting patients for our ongoing national clinical trial. We will soon share the results of an interim analysis of the first 40 CP recipients and PS-matched controls. Once the study is completed, the final results will be published.

The following approvals have been obtained in addition to IRB approvals of the participating centers: MOH Central IRB log No 20-COVID-19-01M (approved on April 2, 2020); SFDA Saudi Clinical Trials Registry No 20041102 (approved on April 14, 2020); and the ClinicalTrials.gov identifier is NCT04347681 (updated and under process).

## Discussion

The study of the Saudi population at risk of severe COVID-19 has some distinct features, as Saudi Arabia was recently challenged by related coronaviruses including MERS-CoV that might lead to distinct immunological responses to SARS-CoV-2. The intention overriding the scientific merit of a randomized controlled trial during this pandemic is an attempt to add CP in the armamentarium against COVID-19, as this readily available agent has historically shown benefit in the treatment of related coronaviruses. There are multiple theoretical and scientific reasons to support our thinking that CP may turn out to be an effective treatment. However, the bottom line is that we do not have enough data to be certain. CP trials that have been published so far have limitations and conflicting data. Our group and others are working hard during this time to establish evidence that could support the therapeutic use of CP for patients with COVID-19.

Limitations of our study include, first, the design of the study, as we could not find it feasible to make the study a randomized controlled trial with our patient population due to the strong perceived benefit of CP in the minds of the patients and treating physicians in this situation. Second, our collaborating centers are of various levels from secondary to tertiary care hospitals with limitation of experience uniformity in conducting such a collaborative study. However, we intend to be a mutually supportive group and keep our study elements uniform. In addition, currently, anti–SARS-CoV-2 antibodies titration facilities are not available countrywide in Saudi Arabia. However, we plan to save aliquots of collected convalescent plasmas to test antibody titers whenever such facilities will be available. Last, we could not include the investigation of some biomarkers like interleukin-6 during treatment in our protocol, due to nonavailability in some centers. Nonetheless, treatment with CP seems promising to treat patients with COVID-19, and a national clinical trial to explore the efficacy and safety of CP is justified.

## Abbreviations

AABB: American Association of Blood Banks
ALC: absolute lymphocyte count
ARDS: acute respiratory distress syndrome
CBAHI: Central Board for Accreditation of Healthcare Institutes
CBC: complete blood count
CE: Conformité Européenne
CP: convalescent plasma
CRP: C-reactive protein
CT: computed tomography
DM: diabetes mellitus
FFP: fresh frozen plasma
HTN: hypertension
ICF: informed consent form
ICU: intensive care unit
IgG: immunoglobulin G
IgM: immunoglobulin M
IRB: institutional review board
MERS-CoV: Middle East respiratory syndrome–related coronavirus
MOH: Ministry of Health
PS: propensity score
PT: partial thromboplastin
rRT-PCR: real-time reverse transcription–polymerase chain reaction
SAE: serious adverse events
SARS-CoV: severe acute respiratory syndrome–related coronavirus
SFDA: Saudi Food and Drug Authority
TACO: transfusion-associated circulatory overload
TBV: total blood volume
TRALI: transfusion-related acute lung injury
WHO: World Health Organization
